# Structural Analysis and Antioxidant Activity of Alkaline-Extracted Glucans from *Hericium erinaceus*

**DOI:** 10.3390/foods13172742

**Published:** 2024-08-29

**Authors:** Zhonghui Qiao, Xiushi Jia, Yuanning Wang, Yuan Wang, Yifa Zhou, Fan Li, Yunhe Qu, Hairong Cheng

**Affiliations:** 1 Engineering Research Center of Glycoconjugates of Ministry of Education, Jilin Provincial Key Laboratory of Chemistry and Biology of Changbai Mountain Natural Drugs, School of Life Sciences, Northeast Normal University, Changchun 130024, China; qiaozh655@nenu.edu.cn (Z.Q.); jiaxs936@nenu.edu.cn (X.J.); wangyn017@nenu.edu.cn (Y.W.); wangy241@nenu.edu.cn (Y.W.); zhouyf383@nenu.edu.cn (Y.Z.); lif885@nenu.edu.cn (F.L.); 2 Institute of Innovation Science & Technology, Central Laboratory, Changchun Normal University, No. 677 North Changji Road, Changchun 130032, China

**Keywords:** *Hericium erinaceus*, polysaccharide, glucan, structural analysis, side chains, antioxidant activity

## Abstract

An alkali-soluble β-glucan (AHEP-A-b, 20 kDa) purified from *Hericium erinaceus* fruiting bodies, was structurally characterized and examined for antioxidant activity. Methylation analysis and NMR spectroscopy show that the backbone of AHEP-A-b is composed of (1→6)-linked-D-β-glucopyran residues, branched at O-3 of glucopyranose (Glc*p*) residues with [→3)-β-D-Glc*p*-(1→] oligosaccharides or single unit of β-Glc*p*. Periodate oxidation analysis and matrix-assisted laser desorption/ionization time of flight mass spectrometry (MALDI-TOF-MS) indicate that the degree of polymerization (DP) of [→3)-β-D-Glc*p*-(1→] side chains is 2 to 8. Functionally, AHEP-A-b is a relatively strong antioxidant as demonstrated by using 2, 2′-azino-bis-(3-ethylbenzthiazoline-6-sulfonic acid) (ABTS) free radical (ABTS^·+^), 1,1-diphenyl-2-picrylhydrazyl (DPPH) radicals, and hydroxyl radicals scavenging assays. The present study lays the foundation for further studies into structure-activity relationships of polysaccharides from *H. erinaceus*.

## 1. Introduction

*Hericium erinaceus* is a wild edible mushroom that has attracted considerable attention due to its high nutritional value [1]. *H. erinaceus* contains a variety of bioactive components, including peptides, fatty acids, sterols, pyrones, etc., with polysaccharides being one of its most important bioactive components [2]. Polysaccharides from *H. erinaceus* have been extracted with water [3], enzymes [4] and ultrasound [5,6]. Even though water extraction is a simple approach, it requires the use of high temperature and has a slow extraction rate [7]. Enzyme-based extraction (usually with cellulase or papain) destroys the cell wall structure and enhances permeability of extracellular membranes, allowing polysaccharides to be solubilized in various solvents that improve their yield [8,9]. Although ultrasonic extraction is fast and efficient [10], the extraction time cannot be too long, because glycosidic bonds will be broken and the yield will be reduced [11].

Studies have shown that (1→3)-β-D-glucan is mainly extracted by using microwave irradiation in water [12]. Fucogalactan and (1→6)-β-D-glucan are mostly extracted from the fruiting bodies of *H. erinaceus* by using hot water [13]. Liao et al [14] reported a novel *H. erinaceus* polysaccharide, that is mostly composed of galactose, glucose and mannose. There are many reports on the overall structure of *H. erinaceus* polysaccharides, but there are relatively few reporting on the structures of the side chains. *H. erinaceus* polysaccharides have considerable potential to decrease serum cholesterol [15], promote gut health [16], treat colon cancer [17], protect against gastric mucosal injury [18], anti-inflammatory [19], decrease triglycerides [20], and induce macrophage proliferation to improve immunity [21].

An antioxidant effect refers to the reduction of oxidative stress that protects cells and prevents disease [22,23] by neutralizing free radicals or inhibiting free radical production to maintain the redox balance. Studies have shown that edible mushroom-derived polysaccharides are excellent natural antioxidants [24]. Various polysaccharides from *Pleurotus ostreatus* [25], *Grifola frondosa* [26,27], and *Agaricus bisporus* [28] show good antioxidant activities. *H. erinaceus* polysaccharides also have good antioxidant activity [29] by scavenging DPPH free radicals [30,31], as well as scavenging hydroxyl radicals, chelating iron, and exhibiting a hepatoprotective capacity [32]. In the present study, we extracted and purified an alkali-soluble β-glucan from *H. erinaceus* and characterized its structure and antioxidant activity.

## 2. Materials and Methods

### 2.1. Materials

Fruiting bodies of *Hericium erinaceus* were purchased from the Zhong Dong Market (Changchun, China) and were identified using rDNA-Internal Transcribed Spacer sequence analysis. The fruiting body is unbranched, with capitate or obovate morphology. The diameter is greater than 5 cm. The spines are pendulous, with length greater than 0.5 cm. All other chemicals were of analytical grade and commercially available.

### 2.2. Extraction and Purification of Polysaccharides

Following water extraction, fruiting bodies from *H. erinaceus* were treated two times with 0.5 M NaOH/0.02% NaBH_4_ solution (1:20 *w*/*v*) at 80 °C for 3 h. HCl (36% *v*/*v*) was then added with stirring until the mixture was neutralized. After centrifugation (4500 rpm, 20 min) of the extract, the supernatant was filtered by hollow fiber ultrafiltration (MWCO: 3000 Da) to remove small molecules and the large amount of NaCl produced by neutralization. The solution was then concentrated under vacuum at 60 °C to a small volume and lyophilized for 12 h to obtain the alkali-soluble *H. erinaceus* polysaccharides (AHEP).

AHEP was then dissolved in distilled water and run through an anion-exchange diethylaminoethyl (DEAE)-cellulose column (8.0 × 20 cm, Cl^−^) that was eluted with distilled water to yield a neutral polysaccharide fraction (AHEP-N) and 0.3 M NaCl to obtain an acidic polysaccharide fraction (AHEP-A). AHEP-A was further purified on a molecular-exclusion chromatography with sepharose gel column (CL-6B, 2.6 cm × 100 cm) that was eluted with 0.15 M NaCl at 0.4 mL/min to yield a homogeneous fraction (AHEP-A-b).

### 2.3. General Methods

The total sugar content was determined by using the phenol-sulfuric acid method, with glucose (Glc) as the standard [33]. 6% phenol solution and concentrated sulfuric acid were added to 1 mL of the sample solution at a concentration of 60 μg/mL. The absorbance value at 490 nm was measured after 30 min, and the sugar content of the sample was calculated by substitute into the standard curve. Uronic acid content was determined by using the colorimetric method, with glucuronic acid (GlcA) as the standard [34]. 0.4 mL of the sample solution at a concentration of 100 μg/mL was added to sulfamic acid-potassium hydroxide solution and concentrated sulfuric acid, boiled in a water and then added m-hydroxybiphenyl-potassium hydroxide solution. The uronic acid content was measured at 525 nm. Protein content was determined using the Bradford assay with bovine serum albumin (BSA) as the standard [35]. 200 μL of the sample solution at a concentration of 0.5 mg/mL added to 1 mL of coomassie brilliant blue solution, and the absorbance value was detected at 595 nm.

### 2.4. Monosaccharide Composition

1 mg of polysaccharide was reacted with 1 mL of hydrochloric acid-methanol solution (2 M) at 80 °C for 16 h, followed by addition of 1 mL 2 M trifluoroacetic acid (TFA) and reacted at 120 °C for 1 h. After derivatization with 1-phenyl-3-methyl-5-pyrazolone (PMP), the hydrolysate was analyzed by using a Shimadzu HPLC system connected to a COSMOSIL 5C18-PAQ column (4.6 mm× 250 mm). The samples were eluted by using 0.1 M phosphate (PBS) with pH = 7 and acetonitrile as mobile phase (*v*/*v* = 81.9:18.1), column temperature chamber was 35 °C, flow rate was 1.0 mL/min, sample injection volume was 10 μL, and UV detection wavelength was 245 nm.

### 2.5. Homogeneity and Molecular Weight Determination

The molecular weight distribution of polysaccharides was determined using high-performance gel permeation chromatography (HPGPC) with a TSK-gel G-3000PWXL (7.8 mm× 300 mm; Tosoh Corporation, Tokyo, Japan) coupled to a HPLC-RID system (Prominence LC-20AT, Shimadzu, Tokyo, Japan). The detection conditions were as follows: the mobile phase was 0.2 M NaCl, the column temperature chamber was 40 °C, the flow rate was 0.6 mL/min, and the sample volume was 20 μL. The calibration curve was established with standard dextrans (80, 50, 25, 12, 5 and 1 kDa).

### 2.6. Ultraviolet (UV) Spectrum Analysis

The UV spectrum was scaned using an UV spectrophotometer (Shimadzu UV-2700, Shimadzu, Tokyo, Japan) over the range of 200 nm to 900 nm. Nucleic acid and protein content were determined by 260 nm and 280 nm.

### 2.7. Fourier Transform Infrared (FT-IR) Spectroscopy

1 mg of polysaccharides were ground with KBr powder and pressed into a tablet for subsequent FT-IR analysis with KBr as a blank control. FT-IR spectra (4000 to 400 cm^−1^) were obtained on a Spectrum Two FT-IR spectrometer (PerkinElmer Inc., Waltham, MA, USA). Data was collected and analyzed using Spectrum Quant (PerkinElmer, Inc., v10.5.2.636) software. 

### 2.8. Methylation Analysis

Methylation analysis was performed according to the procedure of Qu et al [36]. Samples (5 mg) were methylated and acetylated. The resulting partially methylated alditol acetates (PMAA) were analyzed using a gas chromatography-mass spectrometer (GC–MS, 7890B–5977B, Agilent Technologies Inc., Santa Clara, CA, USA) with an DB-35 ms capillary column (30 m × 0.32 mm × 0.25 mm).

### 2.9. NMR Analysis

^1^H, ^13^C, heteronuclear single quantum coherence (HSQC) and heteronuclear multiple bond correlation (HMBC) NMR spectra were performed at 20 °C on a Bruker Avance 600 spectrometer (Bruker, Karlsruhe, Germany) with a Bruker 5 mm broadband probe operating at 600 MHz for ^1^H NMR and 150 MHz for ^13^C NMR. Polysaccharides (20 mg) were dissolved in D_2_O (0.5 mL) and centrifuged (12,000 rpm, 5 min) to remove precipitate. Data were analyzed using standard Bruker software (MestReNova v10.0; Bruker Corporation, Billerica, MA, USA).

### 2.10. Periodate Oxidation and Smith Degradation

Samples (20 mg) were dissolved in NaIO_4_ (10 mL) at a concentration of 15 mM and reacted for 72 h in the dark at 4 °C [37,38]. Ethylene glycol (100 μL) was added to stop the reaction. The solution was then dialyzed (MWCO 1000 Da) for 48 h, and then concentrated and treated with NaBH_4_ (35 mg/mL) for 12 h. After neutralization and dialysis, the solution was lyophilized using lyophilizer (Christ, ALPHA 2-4 LD plus) for 12 h. Dried samples (2 mg) were reacted with 2 mL of 0.5 M trifluoroacetic acid (TFA) at 25 °C for 20 h. TFA was evaporated, and 200 μL of distilled water was added for HPGPC (see Section 2.5 for details) and mass spectrometry analysis.

### 2.11. MALDI-TOF MS

Samples were dissolved in distilled water (5 mg/mL) and assessed by matrix-assisted laser desorption/ionization time of flight mass spectrometry (MADLI-TOF-MS). 10 mg of 2,5-dihydroxybenzoic acid was dissolved in a mixture of 500 μL of acetonitrile and 0.1% trifluoroacetic acid (*v*:*v* = 1:1) to prepare a matrix solution (20 mg/mL). After mixing 1 μL of the sample solution and 1 μL of matrix solution, 1 μL of the mixed solution was placed on a stainless steel target plate and evaporated for 15 min. A BRUKER-Autoflex mass spectrometer in the positive ion mode was used; the scanning range was 100~2000 *m*/*z*. Data were collected using FlexContral 3.4 software and analyzed by FlexAnalysis 3.3.80 software.

### 2.12. Antioxidant Activity Analysis In Vitro

#### 2.12.1. Scavenging Activity on ABTS^·+^ Radicals

The ability of polysaccharidess to scavenge ABTS^·+^ radicals was evaluated as previously reported [39]. Briefly, 50 μL of various concentrations (0.5, 1, 2, 5 and 10 mg/mL) of polysaccharide was added to to 500 μL of ABTS working solution. They were incubated at 37 °C for 30 min in the dark. After centrifugation (12,000 rpm × 5 min), the absorbance of the supernatant was measured at 734 nm (A1). Ascorbic acid was used as the positive control. Distilled water instead of ABTS working solution was used as another control (A2). Distilled water is the blank (A0). The clearance rate of ABTS^·+^ was calculated according to Equation (1):

ABTS^·+^ clearance (%) = [1 − (A1 − A2)/A0] × 100% (1)

#### 2.12.2. Scavenging Activity on DPPH Radicals

The ability to scavenge DPPH radicals was determined as previously reported [40]. DPPH was dissolved in methanol at a concentration of 0.004%. 50 μL of various concentrations (0.5, 1, 2, 5 and 10 mg/mL) of polysaccharide was added to 200 μL of DPPH· solution. The reaction mixture was incubated at 37 °C for 1 h in the dark. Following centrifugation (12,000 rpm × 5 min), the absorption was recorded at 517 nm (A1). The positive control was ascorbic acid. The positive control was ascorbic acid. Methanol instead of the DPPH solution was another control (A2), and distilled water was the blank (A0). The scavenging rate of polysaccharide on DPPH· was calculated according to Equation (2):

Scavenging rate (%) = [1 − (A1 − A2)/A0] × 100%(2)

#### 2.12.3. Scavenging Activity on Hydroxyl Radicals

The ability to scavenge hydroxyl radicals was evaluated as previously reported [41,42]. Briefly, 50 μL of various concentrations (0.5, 1, 2, 5 and 10 mg/mL) of polysaccharide was mixed with 100 μL of FeSO_4_ (9 mM) and 100 μL of salicylic acid (9 mM) (dissolved in ethanol), and then treated with 100 μL of 8.8 mM H_2_O_2_. The mixture was incubated at 25 °C for 30 min. Then the solution was centrifuged (12,000 rpm × 5 min) and the absorbance of the supernatant was measured at 510 nm (A1). The positive control was ascorbic acid. Distilled water instead of H_2_O_2_ was used as another control (A2). Distilled water was the blank (A0). The ability of polysaccharides to scavenge hydroxyl radicals was determined according to Equation (3):

Scavenging rate (%) = [1 − (A1 − A2)/A0] × 100%(3)

### 2.13. Statistical Analysis 

Data are shown as the mean ± standard deviation (SD), and all assessments were from three independent experiments. Data were analyzed for significance using the Student’s *t*-test. Statistical analysis was performed using GraphPad Prism 8.0 software (GraphPad Prism Inc., San Diego, CA, USA). *p* < 0.05 was considered statistically significant.

## 3. Results and Discussion

### 3.1. Preparation of Polysaccharides

**Figure 1:**
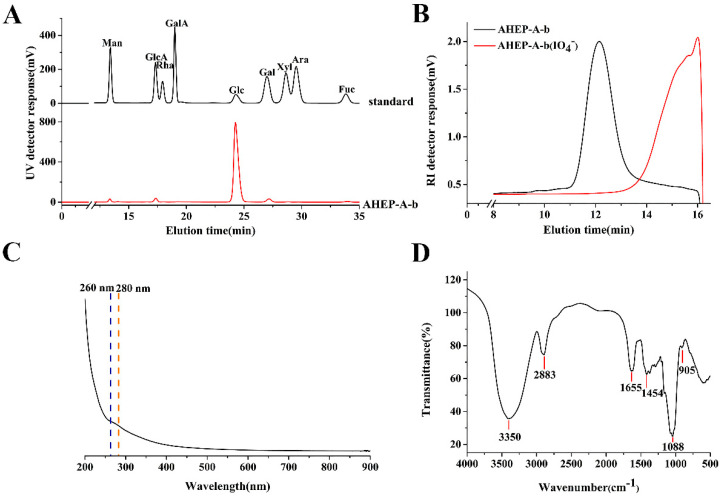
Determination of physicochemical properties of polysaccharides. (**A**) Monosaccharide composition of AHEP-A-b. (**B**) Elution profile of AHEP-A-b and AHEP-A-b (IO_4_^−^) using high-performance gel permeation chromatography. (**C**) Ultraviolet (UV) spectrum of AHEP-A-b. (**D**) Fourier transform infrared (IR) spectrum of AHEP-A-b. AHEP, alkali-soluble *H. erinaceus* polysaccharide; A, acidic polysaccharide fraction; A-b, homogenous fraction; AHEP-A-b (IO_4_^−^), Periodate oxidation and Smith degradation products of AHEP-A-b; RI, refractive index.

AHEP was extracted from the fruiting bodies of *H. erinaceus* following water extraction. The yield was 7.6% relative to the dry weight of the material, and the total sugar content was 86.7%, the uronic acid content was 1.0%, and the protein content was 4.8%. AHEP was separated into AHEP-N and AHEP-A, and the homogeneous fraction AHEP-A-b was obtained from AHEP-A with a yield of 17.1% relative to AHEP. Monosaccharide composition indicated that this polysaccharide is composed of 90.1% glucose, 2.8% galactose, 1.5% mannose, 4.4% glucuronic acid and 1.2% fucose (Figure 1A). The molecular weight of AHEP-A-b is ~20 kDa as determined by HPGPC (Figure 1B).

### 3.2. Ultraviolet (UV) and FT-IR Spectra

As shown in Figure 1C, UV absorption peaks were absent at 260 nm and 280 nm, indicating that AHEP-A-b does not contain nucleic acids or proteins and is a high-purity polysaccharide.

The FT-IR spectrum of AHEP-A-b is shown in Figure 1D. The broad and strong absorption peak at 3350 cm^−1^ results from the stretching vibrations of O-H groups, while the peak at 2883 cm^−1^ arises from C-H stretching vibrations and the stronger signal at 1655 cm^−1^ is associated with bound water and C=O groups which confirms the presence of GlcA. The wavenumber of 1454 cm^−1^ results from variable-angle vibrations of C-H groups, and the peak at 1088 cm^−1^ is attributed to the pyranose ring [43]. The peak at 905 cm^−1^ indicates the presence of β-linked glycosyl residues [44].

### 3.3. Methylation Analysis

**Figure 2:**
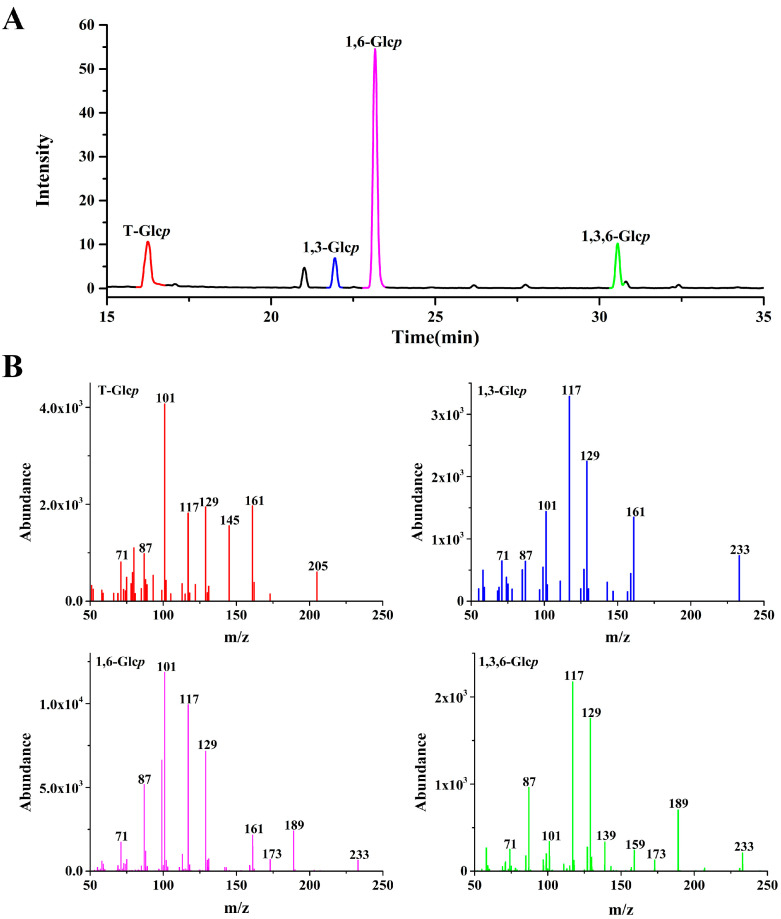
Methylation analysis of *H. erinaceus* polysaccharide homogenous fraction (AHEP-A-b) as determined by GC-MS. (**A**) The total ion chromatogram profiles of partially methylated alditol acetates (PMAAs). (**B**) The ion fragments of partially methylated alditol acetates (PMAAs).

Methylation analysis was performed to determine the glycosidic linkage in AHEP-A-b, which contains a minimal amount of glucuronic acid that was not reduced in advance. AHEP-A-b was methylated, hydrolyzed and acetylated, followed by GC–MS analysis to deduce the amount of alditol acetate. The total ion chromatogram profiles and ion fragments of partially methylated alditol acetates (PMAAs) are shown in Figure 2. Table 1 shows that the glycosidic linkage of glucose in AHEP-A-b is in various forms, i.e., (1→6)-linked, (1→3,6)-linked, (1→3)-linked and terminal-linked. The (1→6)-linked (69.8%) and (1→3,6)-linked (11.7%) forms indicate that the backbone of AHEP-A-b is (1→6)-glucan, branched at O-3 (14.4%). The terminal glucose residue (10.3%) and (1→3)-linked glucose (8.2%) may be present as side chains linked to the backbone.

### 3.4. NMR Structural Analysis

**Figure 3:**
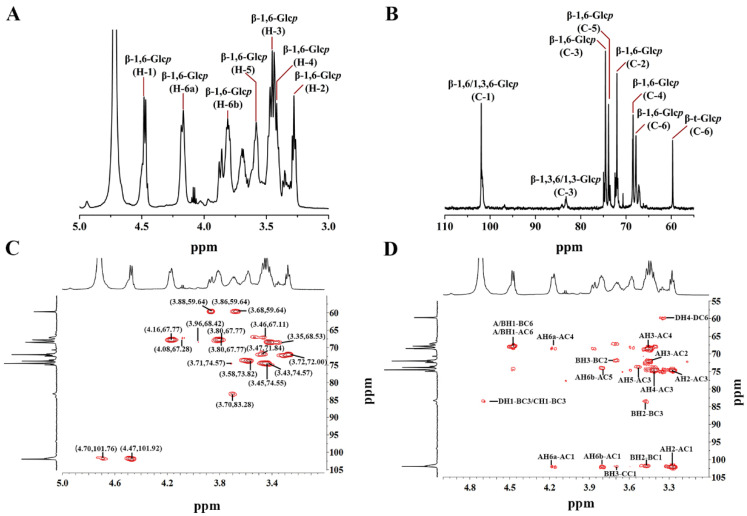
NMR spectra of *H. erinaceus* polysaccharide AHEP-A-b. (**A**) ^1^H NMR spectrum. (**B**) ^13^C NMR spectrum. (**C**) HSQC spectrum. (**D**) HMBC spectrum.

1D/2D NMR spectra of AHEP-A-b are shown in Figure 3, with chemical shifts listed in Table 2. In the ^1^H-NMR spectrum (Figure 3A), anomeric proton peaks at 4.47 ppm and 4.70 ppm, respectively, are associated with [→6)-β-D-Glc*p*-(1→/→3,6)-β-D-Glc*p*-(1→ and →3)-β-D-Glc*p*-(1→/β-D-Glc*p*-(1→]. The signals at 4.16 ppm and 3.80 ppm are attributed to H-6a and 6b of [→6)-β-D-Glc*p*-(1→]. In the ^13^C-NMR spectrum (Figure 3B) of AHEP-A-b, the six most obvious resonances at 101.92 ppm, 72.00 ppm, 74.55 ppm, 68.39 ppm, 73.82 ppm, and 67.77 ppm, are attributed to C-1, C-2, C-3.C-4, C-5 and C-6 groups in (1→6)-linked-D-β-glucopyran residues. The weak signal at 83.28 ppm is associated with the C-3 of [→3,6)-β-D-Glc*p*-(1→/→3)-β-D-Glc*p*-(1→], and the signal at 59.62 ppm is assigned to C-6 of β-D-Glc*p*-(1→/→3)-β-D-Glc*p*-(1→].

Other proton and carbon resonances are shown in the HSQC spectrum (Figure 3C). The strong signals at 4.47/101.92 ppm, 3.45/74.55 ppm and 4.16; 3.80/67.77 ppm arise from H1/C1, H3/C3 and H6/C6 signals of [→6)-β-D-Glc*p*-(1→], whereas the strong H1/C1 signal at 4.47/101.92 ppm, the H3/C3 signal at 3.70/83.28 ppm and the H6/C6 signal at 3.53;3.46/67.11 ppm are attributed to [→3,6)-β-D-Glc*p*-(1→]. The peaks at 4.70/101.76 and 3.88;3.68/59.64 ppm arise from H1/C1 and H6/C6 groups of the terminal-β-D-Glc*p* residue.

Sugar residues in [→6)-β-D-Glc*p*-(1→, →3,6)-β-D-Glc*p*-(1→, →3)-β-D-Glc*p*-(1→ and β-D-Glc*p*-(1→] are labeled as A, B, C and D, respectively. In the HMBC spectrum (Figure 3D), anomeric proton and carbon peaks of glycosyl residues AH6a/AC1 and AH6b/AC1 suggest that [→6)-β-D-Glc*p*-(1→] are connected to each other. Based on the coupled signals of A/BH1/BC6, A/BH1/AC6, and C/DH1/BC3, we decided that [→6)-β-D-Glc*p*-(1→] is connected to [→3,6)-β-D-Glc*p*-(1→], whereas [→3)-β-D-Glc*p*-(1→/β-D-Glc*p*-(1→] is connected to the C-3 of [→3,6)-β-D-Glc*p*-(1→].

Overall, our NMR results indicate that the backbone of AHEP-A-b is composed of [→6)-β-D-Glc*p*-(1→], with side chains of [→3)-β-D-Glc*p*-(1→ and β-D-Glc*p*-(1→] linked to the main chain at the C-3 position of Glc*p*.

### 3.5. Periodate Oxidation

Periodate oxidation and MALDI-TOF-MS were performed to determine the length of [→3)-β-D-Glc*p*-(1→] side chains in this (1→6)-β-D-glucan. Periodate oxidation and Smith degradation of AHEP-A-b lead to the degradation product AHEP-A-b (IO_4_^−^). The Changes in molecular weight before and after the reaction are shown in Figure 1B. Following degradation, HPGPC chromatographic peaks of the polysaccharides disappeared, whereas those of the derivatives appeared as the molecular weight decreased. The trihydroxyl group in the backbone of [→6)-β-D-Glc*p*-(1→] was removed during the reaction. The Glc*p* residue [→3,6)-β-D-Glc*p*-(1→] linked to the C-3 position in the main chain, as well as [→3)-β-D-Glc*p*-(1→] appended side chains could not be oxidized by periodate due to the absence of a dihydroxy group, thus leading in production of oligosaccharide derivatives.

**Figure 4:**
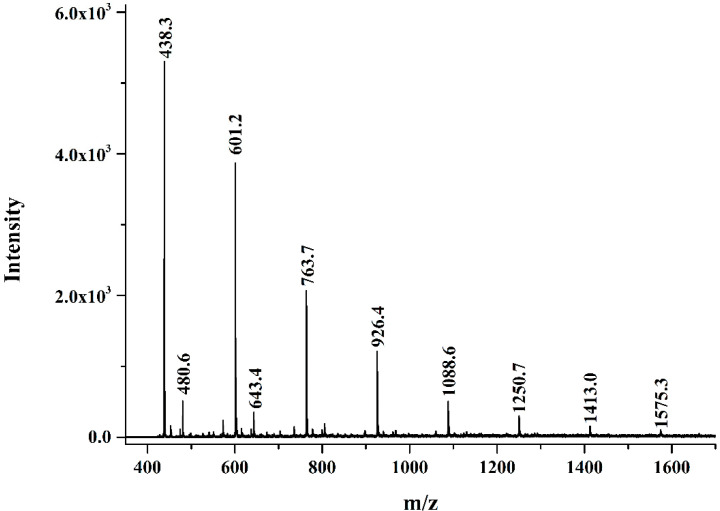
MALDI-TOF-MS of periodate oxidation and Smith degradation products of *H. erinaceus* polysaccharide AHEP-A-b (IO_4_^−^).

MALDI-TOF-MS of the degradation product AHEP-A-b (IO_4_^−^) (Figure 4) showed mass-to-charge (m/z) ratios at 439, 601, 763, 925, 1087, 1249, 1411, and 1573. These molecular ion peaks were calculated and assigned, and represent the sum of peaks arising from adipyose and sodium ions with glycerol [Hex_2_+C_3_H_8_O_3_+Na^+^], from hexanetrinose with glycerol and sodium ions [Hex_3_+C_3_H_8_O_3_+Na^+^], from hexametetranose with glycerol and sodium ions [Hex_4_+C_3_H_8_O_3_+Na^+^], from hexanepentase with glycerol and sodium ions [Hex_5_+C_3_H_8_O_3_+Na^+^], from hexose and sodium with glycerol [Hex_6_+C_3_H_8_O_3_+Na^+^], from hexanose with glycerol and sodium [Hex_7_+C_3_H_8_O_3_+Na^+^], from hexaneschanose with glycerol and sodium [Hex_8_+C_3_H_8_O_3_+Na^+^], and from hexanese and sodium ion with glycerol [Hex_9_+C_3_H_8_O_3_+Na^+^]. The corresponding structure was determined to arise from [→3)-β-D-Glc*p*-(1→] side chains of Glc*p* with its C-3 linked to the (1→6)-β-D-glucan backbone and polymerization degrees from 2 to 8.

Periodate oxidation combined with Smith degradation is a chemical method used to determine whether [→3)-β-D-Glc*p*-(1→] arises from the backbone or branched side chains. Using this approach, various (1→3)-β-D-glucans prepared from edible fungi have been identified, such as *Flammulina velutipes* [45], *Pleurotus florida* [46], *Pleurotus citrinopileatus* [47] and *Ramaria botrytis* [48].

### 3.6. Antioxidant Activity

**Figure 5:**
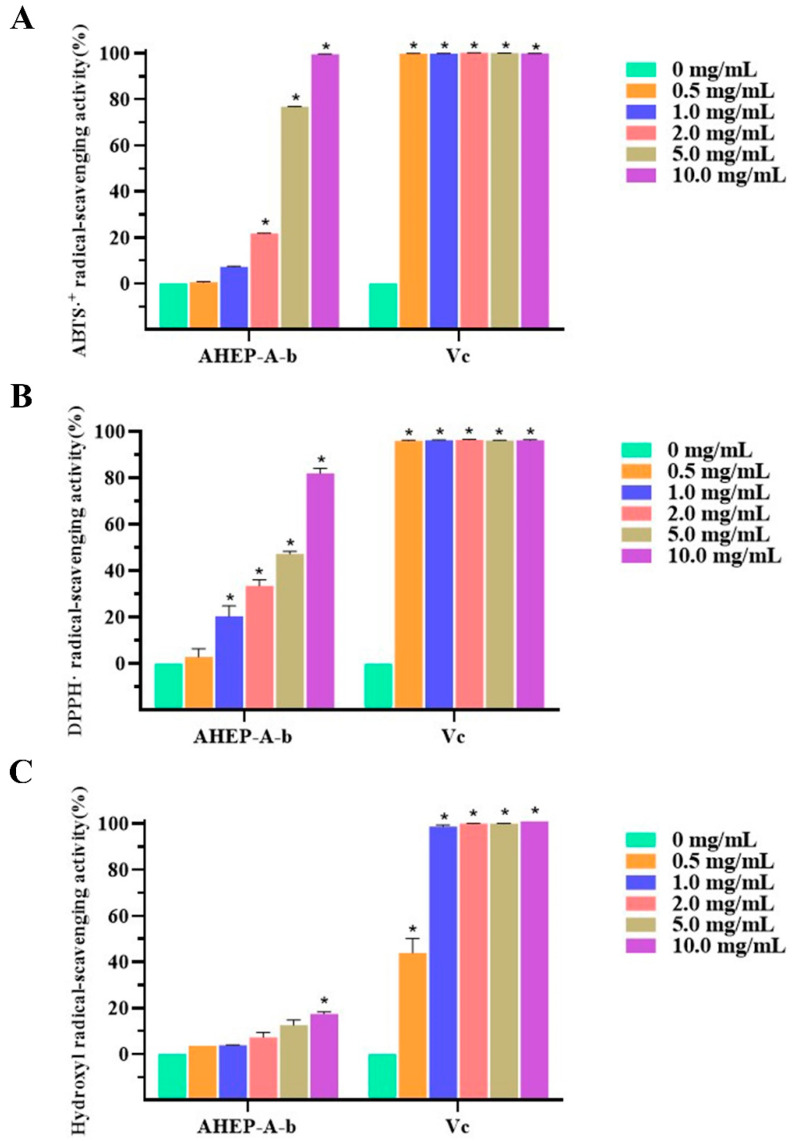
Antioxidant activity of AHEP-A-b. (**A**) Scavenging ABTS^·+^ radical. (**B**) Scavenging DPPH·. (**C**) Scavenging hydroxyl radical (•OH-). Vc is the positive control. Data are shown as the mean ± SD, (n = 3, *: *p* < 0.05). All experiments were performed in triplicate.

AHEP-A-b antioxidant activity was assessed by using ABTS^·+^, DPPH· and hydroxyl scavenging assays (Figure 5). At a AHEP-A-b concentration of 10 mg/mL, the clearance rate for ABTS^·+^ was ~99.6%, which is close to that of the positive control. Over the concentration range of 0.5 mg/mL to ~10 mg/mL, the ability of AHEP-A-b to scavenge ABTS^·+^ was enhanced as the concentration increased. 

The DPPH·scavenging effect of AHEP-A-b increased as the concentration was increased from 0.5 to 10 mg/mL. At 10 mg/mL, the clearance rate was greatest, albeit lower than that of the positive control (96.2%). There was no significant difference in the clearance rate of hydroxyl radicals over the concentration range of 0.5 mg/mL to 5 mg/mL compared with the blank control. At 10 mg/mL, however, AHEP-A-b to significantly scavenge hydroxyl·, with a clearance rate of 17.5%. assays, IC50 values from the three radical scavenging assays were 3.03 ± 0.05 mg/mL, 3.90 ± 0.23 mg/mL and 89.15 ± 1.00 mg/mL, respectively. These results demonstrated that AHEP-A-b has antioxidant activity. Some reports state that the antioxidant activity of edible fungi is closely related to that of β-glucan [49]. Similar results were obtained in the study of polysaccharides derived from the mycelium of *H. erinaceus* [32], with the higher fraction of Glc*p* having a greater ability to scavenge free radicals. ABTS^·+^ and DPPH· have been widely used to evaluate the antioxidant activity of natural products [50,51]. Previous reports showed that ABTS^·+^assay may be more useful than DPPH· assay for detecting antioxidant capacity of a variety of foods [52]. However, our results showed that the two methods exerted similar activity in detecting antioxidant activity of polysaccharides. It is noteworthy that inhibition of hydroxyl radical (OH.) of AHEP-A-b is relative insensitive. All these results suggest that AHEP-A-b have different scavenging efficiencies against different ROS, laying a theoretical foundation for their potential applications. To comprehensively analyze the antioxidant activity of the polysaccharide, more different free radicals will be applied in the future study.

## 4. Conclusions

An alkali-soluble β-glucan (AHEP-A-b) was purified from alkali-extracted polysaccharides from *Hericium erinaceus* by using anion exchange and gel-permeation chromatographies. Structural analysis showed that the backbone of AHEP-A-b is a (1→6)-β-D-glucan, branched at O-3 of Glc*p* with [→3)-β-D-Glc*p*-(1→] short chains or single-units of β-Glc*p*. Periodate oxidation and MALDI-TOF-MS indicated that the degrees of polymerization side chains [→3)-β-D-Glc*p*-(1→] present in this (1→6)-β-D-glucan ranges from 2 to 8. Functionally, AHEP-A-b exhibits antioxidant activity as assessed by ABTS^·+^, DPPH· and hydroxyl scavenging assays. 

## Figures and Tables

**Table 1:** Linkages type analysis of *H. erinaceus* polysaccharide homogenous fraction AHEP-A-b as determined by GC-MS.

Methylated Sugars	Linkages	Molar (%)	Mass Fragments (*m*/*z*)
2,3,4-Me_3_-Glc*p*	1,6-	69.8	101, 117, 129, 161, 173, 189, 233
2,4-Me_2_-Glc*p*	1,3,6-	11.7	117, 129, 159, 189, 233, 261, 305
2,4,6-Me_3_-Glc*p*	1,3-	8.2	101, 117, 129, 161, 189, 233, 277
2,3,4,6-Me_4_-Glc*p*	t-	10.3	101, 117, 129, 145, 161, 205

**Table 2:** Chemical shift assignments of ^1^H and ^13^C signals for *H. erinaceus* polysaccharide homogenous fraction AHEP-A-b.

Linkage Type	Chemical Shift (ppm)					
	C-1/H-1	C-2/H-2	C-3/H-3	C-4/H-4	C-5/H-5	C-6/H-6
(A)→6)-β-D-Glc*p*-(1→	101.92/4.47	72.00/3.27	74.55/3.45	68.39/3.43	73.82/3.58	67.77/4.16; 3.80
(B)→3,6)-β-D-Glc*p*-(1→	101.92/4.47	71.84/3.47	83.28/3.70	68.53/3.35	74.22/3.83	67.11/3.53; 3.46
(C)→3)-β-D-Glc*p*-(1→	101.76/4.70	72.00/3.27	83.28/3.70	68.44/3.41	74.22/3.83	59.51/3.86; 3.79
(D)→β-D-Glc*p*-(1→	101.76/4.70	71.84/3.47	74.57/3.71	68.55/3.58	73.82/3.58	59.64/3.88; 3.68

## Data Availability

The original contributions presented in the study are included in the article, further inquiries can be directed to the corresponding authors.
